# Kuramoto model simulation of neural hubs and dynamic synchrony in the human cerebral connectome

**DOI:** 10.1186/s12868-015-0193-z

**Published:** 2015-09-02

**Authors:** Ruben Schmidt, Karl J. R. LaFleur, Marcel A. de Reus, Leonard H. van den Berg, Martijn P. van den Heuvel

**Affiliations:** Department of Neurology, Brain Center Rudolf Magnus, University Medical Center Utrecht, Heidelberglaan 100, PO Box 85500, 3508 GA Utrecht, Netherlands; Department of Psychiatry, Brain Center Rudolf Magnus, University Medical Center Utrecht, Heidelberglaan 100, PO Box 85500, 3508 GA Utrecht, Netherlands

**Keywords:** Hub node, Structural connectivity, Neural synchronization, Cortical coupling, Suppression, Perturbation

## Abstract

**Background:**

The topological structure of the wiring of the mammalian brain cortex plays an important role in shaping the functional dynamics of large-scale neural activity. Due to their central embedding in the network, high degree hub regions and their connections (often referred to as the ‘rich club’) have been hypothesized to facilitate intermodular neural communication and global integration of information by means of synchronization. Here, we examined the theoretical role of anatomical hubs and their wiring in brain dynamics. The Kuramoto model was used to simulate interaction of cortical brain areas by means of coupled phase oscillators—with anatomical connections between regions derived from diffusion weighted imaging and module assignment of brain regions based on empirically determined resting-state data.

**Results:**

Our findings show that synchrony among hub nodes was higher than any module’s intramodular synchrony (*p* < 10^−4^, for cortical coupling strengths, *λ*, in the range 0.02 < *λ* < 0.05), suggesting that hub nodes lead the functional modules in the process of synchronization. Furthermore, suppressing structural connectivity among hub nodes resulted in an elevated modular state (*p* < 4.1 × l0^−3^, 0.015 < *λ* < 0.04), indicating that hub-to-hub connections are critical in intermodular synchronization. Finally, perturbing the oscillatory behavior of hub nodes prevented functional modules from synchronizing, implying that synchronization of functional modules is dependent on the hub nodes’ behavior.

**Conclusion:**

Our results converge on anatomical hubs having a leading role in intermodular synchronization and integration in the human brain.

**Electronic supplementary material:**

The online version of this article (doi:10.1186/s12868-015-0193-z) contains supplementary material, which is available to authorized users.

## Background

The human brain has to continuously process a large variety of information relating to vision, hearing, motor function, and numerous associative processes. The topological organization of the brain’s wiring pattern has an important role in enabling complex functionality, evidenced by studies examining structural wiring architecture of mammalian and nematode neural systems that revealed several network attributes of an efficient processing and communication structure [1, 2]. Neural systems have been shown to exhibit synchrony patterns associated with communication between segregated anatomical neural components in terms of neuronal discharges, beta and gamma band fluctuations and resting-state blood oxygenation level dependent (BOLD) signals of brain regions [3–9]. Synchronized activity of specialized groups of neurons has been proposed to provide a neural basis for binding and integrating information [10–12]. Moreover, brain disorders such as schizophrenia, epilepsy, autism and Alzheimer and Parkinson disease have been associated with abnormal neural synchronization [7].

These observations of (dys)synchrony called for the formulation of mathematical models to provide insight into the neural dynamics and its relation to the complex architecture of the underlying structural pathways [13, 14].

Embracing network science as a general mathematical framework to understand topological organizational features of neural wiring, studies have revealed high levels of clustering and community structure, suggesting the existence of functional sub-systems, as well as short communication pathways and the existence of high-degree hub nodes that take a central role in the overall network organization [3, 15–18]. Furthermore, recent research has shown that a highly interconnected collection of high-degree nodes, the ‘rich club’, is embedded across many functional domains and may thus serve as an anatomical infrastructure for global neural communication between otherwise segregated systems [1, 2, 16, 19, 20].

Using the Kuramoto model [21] to simulate brain interactions through synchrony on the basis of structural connectivity, functional implications of the organization of brain connectivity can be examined. These include for example how cross-domain integration can be influenced by the underlying wiring anatomy of the brain. Simulation studies employing the Kuramoto model have investigated synchronization patterns in the cortical brain networks of the cat [3], the macaque [22] and the human [23–25], showing correspondence with resting-state functional data supportive of the applicability of the model [24, 26]. Here, extending to previous work on random or targeted attack in structural brain networks [22, 27, 28], we studied the synchronization effects of suppressed structural connectivity and perturbed intrinsic oscillatory behavior of brain regions in the human cortical network. Simulating these phenomena we shed light on the role of anatomical hubs in functional dynamics of the brain network, which may ultimately contribute to the understanding of effects of structural brain pathology on brain function.

## Methods

### Subjects

The cohort consisted of the 40 healthy subjects (mean age ± standard deviation 27 ± 6.9; male/female: 27/13) previously reported on by Van den Heuvel et al. and used as a replication set in [29]. Each subject gave informed written consent according to the Declaration of Helsinki and the study was approved by the medical ethics committee for research into humans of the University Medical Center Utrecht. Scanning sessions lasted 45 min and were performed on a 3 Tesla Philips Achieva Clinical scanner. For each subject, diffusion weighted imaging data (acquisition parameters: SENSE-p = 3; two sets of 30 different weighted directions, and 2 × *b* = *0* images; repetition time (TR)/echo time (TE) = 7,035/68 ms, 2 × 2 × 2 mm, 75 slices covering whole brain, *b* weighting of 1000 s/mm^2^, second set with reversed *k*-space readout) and an anatomical T1 image for anatomical reference (3D fast field echo (FFE) using parallel imaging; TR/TE = 10 ms/4.6 ms; field of view (FOV) = 240 × 240 mm, 200 slices covering whole brain, 0.75 × 0.75 × 0.75 mm) were acquired.

### Connectome reconstruction

The structural network from [29] was recomputed at a 219 × 219 network resolution using a subdivision of the Desikan–Killiany atlas [30]. Each area of the brain was designated as a node in the network, and edges represent anatomical connections between these nodes. For each individual, if reconstructed white matter fiber(s) were found between two nodes *i* and *j*, then a 1, indicating the presence of a connection, was placed in cell *c*(*i*,*j)* of the 219 × 219 connectivity matrix, *M*. If no fibers were found connecting regions *i* and *j*, then these nodes were taken to be non-connected and the corresponding entry in the weighted connectivity matrix *M* was set to zero. From the individual connectivity matrices a binary group matrix was constructed having a 1 for connections present in at least 40 % of the subjects and a 0 otherwise [31].

### Cortical network, rich club, and functional modules

A template of resting-state networks (RSNs) [32–35], distinguishing between subnetworks of regions that show correlated activity during rest based on resting-state functional MRI (fMRI) data from a group of healthy subjects, was used to define 11 commonly reported RSN networks (Additional file 1: Figure S1) [16]. Van den Heuvel and Sporns 2013 used Independent Component Analysis (ICA) decomposition on the resting-state fMRI data to define this RSN template. The 11 functional network maps were realigned to the group-averaged normalized T1 images of the subjects and resampled to the 219 parcellation scheme of the diffusion data. As performed in the Van den Heuvel and Sporns paper [16], each node was assigned a single RSN by means of a majority vote based on its voxels’ participation across the 11 RSNs. This resulted in each network node participating in a single functional module, with the functional modules varying in size from 11 to 31 nodes. Following the line of thought of previous studies [32, 33], these 11 RSNs were taken as a description of 11 functional domains (i.e. modules) of the human brain.

Hub nodes were selected on the basis of degree of connectivity. Consistent with the size and layout of a neural rich club as reported in literature [2, 3, 36], the thirty-nine nodes with the highest number of connections (17.8 % of all nodes) were designated as members of the rich club. In concurrence with previous findings, the anatomical rich club included the insula, precuneus, superior frontal and superior parietal regions and participated in all of the 11 functional modules [16, 20].

In order to verify our findings on the human cortical network, a reconstruction of the macaque cortical network was used based on collated data from tract-tracing experiments. To this end the open source CoCoMac neuroinformatics database [37] was queried for white matter axonal projections linking cortical regions of the Felleman and Van Essen 91 atlas [38]. The Felleman and Van Essen 91 atlas describes a single hemisphere and includes a parcellation of the cerebral cortex into 78 non-overlapping regions. For all node pairs a 1 was included in the symmetric structural connectivity (SC) matrix if the query returned at least 1 report and if of these reports at least 40 % were positive reports; otherwise a 0 was included in the matrix. The resulting SC matrix had a 27.6 % density.

Since no predefined functional modules were available for the macaque, modules were created based on structural connectivity data by maximizing the number of within-group edges [39]. Modules were selected so that each module contained a minimum of 8 nodes out of the total of 78 nodes.

### The Kuramoto model

**Fig. 1:**
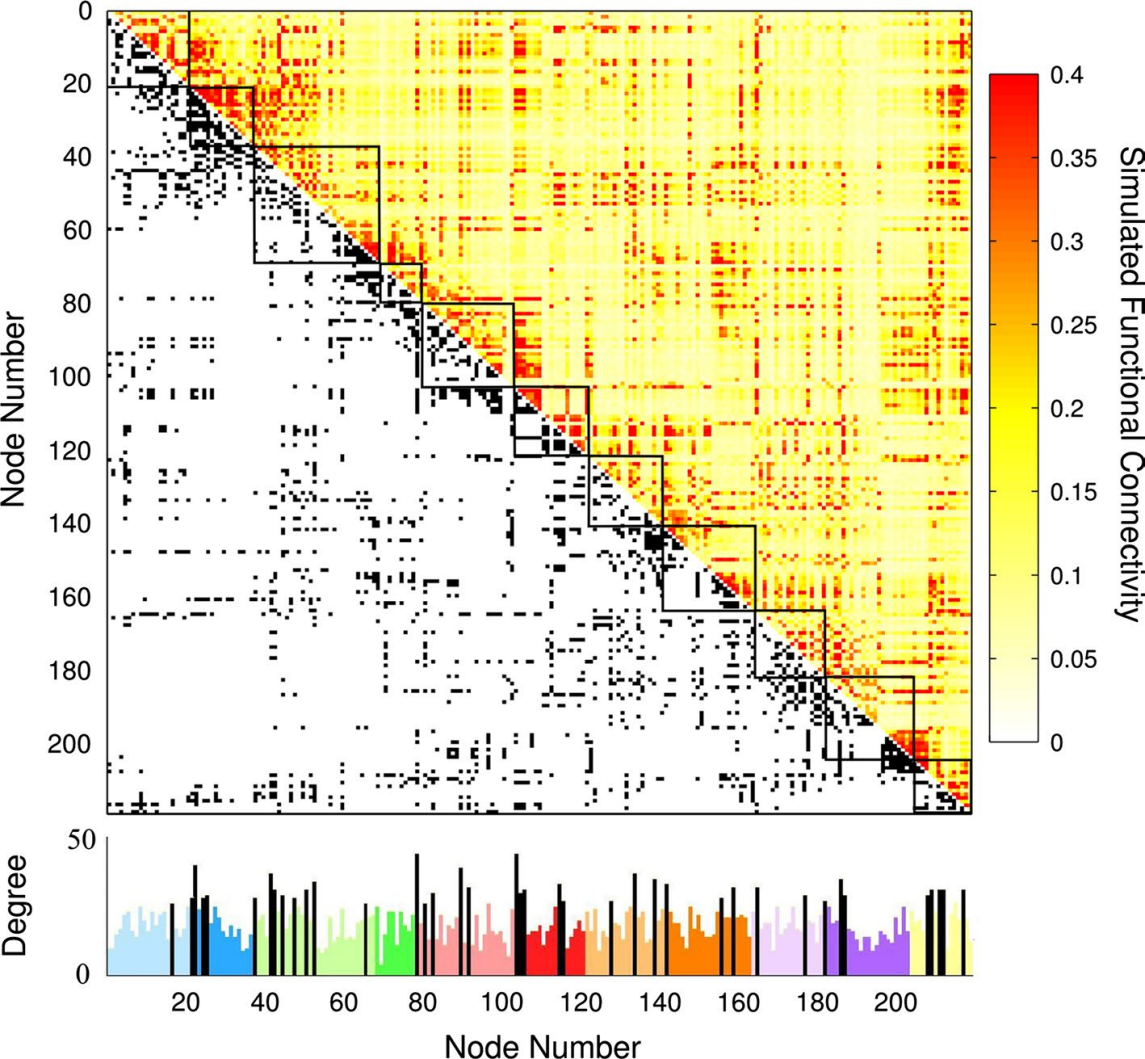
Structural binary input with functional output. The binary undirected adjacency matrix that describes the cortical brain network in the *lower triangle*, and the edgewise weighted synchronization output (simulated functional connectivity) in the *upper triangle* (generated at cortical coupling factor λ = 0.02). The histogram at the *bottom* shows the degree (i.e. number of connections) of each node. Each module is color-coded and rich club nodes, distributed across the functional modules, are shown in *black*. The eleven functional modules are: (1) Default Mode, (2) Primary Visual, (3) Extrastriate Visual, (4) Motor, (5) Sensory, (6) Bilateral Parietal, (7) Left Parietal Frontal, (8) Right Parietal Frontal, (9) Auditory, (10) Salience, and (11) Frontal

The Kuramoto model simulates dynamic behavior of a set of coupled oscillators. Applying the Kuramoto model to the group-averaged anatomical brain network, each node was assigned an oscillator with a fixed, random internal angular frequency and an initial random phase. The model is defined as,

1 $$ \dot{\theta }_{i} = \omega_{i} + \lambda \mathop \sum \nolimits_{{{\text{j = }}1}}^{\text{N}} {W}_{ji} \sin (\theta_{j} - \theta_{i} ) $$

where *θ*_*i*_*(t)* and *ω*_*i*_ are the phase and internal angular frequency of oscillator *i* respectively. The cortical coupling strength that is applied to the edges is denoted by *λ*. *W*_*ij*_ is the symmetrical group matrix containing all connections between cortical nodes (Fig. 1) and *N* is the total number of nodes. The set of differential equations (Eq. 1) was solved numerically using a Runge–Kutta solver. During every run of the model, each node was assigned a random initial phase, *θ*, uniformly distributed between [−π, π] and a random internal frequency, *ω*, uniformly distributed between [0, 1]. Simulations were carried out for T = 700 with a transient time of τ = 300, keeping these parameters the same as in previous work [3]. Explorative longer runs of T = 5000 did not change the nature of our findings. Output data from the last 400 time points was then used in the analyses.

### Synchronization metrics and computational details

Two order parameters *r* and *r*_link_ were used to describe global dynamic coherence.^1^ The first order parameter, *r*, is based on a complex number *z* defined as,

2 $$ z\left( t \right) = \frac{1}{N}\sum\nolimits_{j = 1}^{N} {e^{{i\theta_{j} (t)}} } = r\left( t \right)e^{i\varPhi (t)} $$

where *r(t)* is the modulus of *z(t)*. The variable *r(t)* is a measure of the phase synchrony among the population of *N* oscillators. *Φ(t)* is the average combined phase of all oscillators. Averaging *r(t)* over time yields the first order parameter, *r*.

In addition to the average phase coherence, *r*, the average edgewise synchrony also provides information about the state of the network [3, 40]. The edgewise synchrony between nodes *i* and *j*, C_*ij*_, is defined by

3 $$ C_{ij} = \frac{1}{\Delta t}\left| {\sum\nolimits_{\tau }^{\tau + \Delta t} {e^{{i[\theta_{i} \left( t \right) - \theta_{j} \left( t \right)]}} } } \right| $$

together forming *C*, the simulated functional connectivity matrix. Average edgewise synchronization levels are taken together to yield r_link_,

4 $$ r_{\text{link}} = \frac{1}{N(N - 1)}\sum\nolimits_{i,j} {C_{ij} } $$

where *N* is the total number of nodes of the network. The phase coherence, *r,* and the fraction of synchronized node pairs, *r*_link_, together describe the global dynamics of the system.

Going beyond the examination of global dynamics, synchronization dynamics were also studied on a modular level, analyzing nodes together on the basis of their functional module assignment. In order to assess the likelihood of particular nodes to be in synchrony, node pairs need to be classified as either synchronized or non-synchronized. To this end the *r*_link_ parameter, ranging from 0 (total incoherence) to 1 (perfect synchrony), was used to define the proportion of nodes that are in synchrony. Assuming a threshold level of synchronization, *C*_*ij*_, exists above which the nodes’ oscillatory behavior converge to full synchrony, a theoretically equivalent system with equal order parameter *r*_link_, was constructed composed of *N*(*N*-1) × *r*_link_ perfectly coherent and *N*(*N*-1) × (1-*r*_link_) completely incoherent node pairs, described by a binary synchronization matrix *F*_*ij*_:

5 $$ F_{ij} = \left\{ {\begin{array}{*{20}l} {1,\quad N\left( {N - 1} \right)r_{\text{link}} \,\,{\rm largest\,\,elements\,\,of}\, C_{ij} } \\ {0,  \quad {\rm{lower\,\,values\,\,of}}\, C_{ij} } \\ \end{array} } \right. $$

The number of edges present in *F* now matches the number of synchronized connections predicted by *r*_link_. The node pairs showing the highest synchronization levels are thus categorized as synchronized, and those with lower synchrony as unsynchronized. In order to study synchronization levels independent of initial conditions, *n* = 10^3^ trials were run for each cortical coupling strength *λ*. The *F*_*ij*_ values were averaged across trials into *r*_*ij*_ such that,

6 $$ r_{ij} = \frac{1}{n}\sum\nolimits_{l = 1}^{n} {F_{ij} (l)} $$

where *r*_*ij*_ reflects the probability of regions *i* and *j* to be synchronized and where *l* is the trial number. Based on the probability of synchronization between any two areas, modular synchronization, *r*_*αβ*_, is calculated as,

7 $$ r_{\alpha \beta } = \frac{1}{{N_{\alpha } N_{\beta } }}\sum\nolimits_{i \in \alpha ,\,j \in \beta } {r_{ij} } $$

where *i* describes the nodes in module *α*, and *j* are nodes in module *β*. *N*_*α*_ and *N*_*β*_ are the number of nodes in module *α* and *β* respectively. In the case of intramodular synchrony (*α* = *β*), Eq. 7 was slightly adapted to Eq. 8 so that synchrony within a node (i.e. *r*_*ii*_) was eliminated, to avoid module size to have an effect on intramodular synchrony.

8 $$ r_{\alpha \alpha } = \frac{1}{{N_{\alpha } (N_{\alpha } - 1)}}\sum\nolimits_{i,j \ne i \in \alpha } {r_{ij} } $$

The mean field representation is an alternative representation of the Kuramoto model, describing each oscillator’s dynamics by a centroid with a mean radius and a mean phase:

9 $$ \dot{\theta }_{k} = \omega_{k} - R_{k} \cdot \sin \,(\phi_{k} - \theta_{k} ) $$

derived by substituting the following expression into the original Kuramoto model definition of Eq. 1 [21, 41]:

10 $$ R_{k} e^{{i\phi_{k} }} = \frac{1}{{D_{k} }}\sum\nolimits_{j} {W_{kj} e^{{i\theta_{j} }} } $$

where *R*_*k*_ is the radius and *ϕ*_*k*_ the phase of the centroid representing node *k* and *D*_*k*_ is the degree of node *k* (i.e. the number of other nodes it is connected to). The centroid’s radius *R*_*k*_ captures the phase coherence among the nodes that node *k* is connected with and, as can be seen from Eq. 9, it denotes the strength by which the mean field influences the frequency of node *k*. Rewriting Eq. 10 as follows yields each module’s contribution to the mean field and therefore one obtains a measure of how strongly each of the modules influence the frequency of node *k*:

11 $$ \frac{1}{{D_{k} }}\sum\nolimits_{j} {W_{kj} e^{{i\theta_{j} }} } = \frac{1}{{D_{k} }}\left[ {\sum\nolimits_{j\in \alpha } {W_{kj} e^{{i\theta_{j} }} } + \sum\nolimits_{j\in \beta } {W_{kj} e^{{i\theta_{j} }} } + \ldots  } \right] = \frac{1}{{D_{k} }}\left[ {D_{k,\alpha } R_{k,\alpha } e^{{i\phi_{k,\alpha } }} + D_{k,\beta } R_{k,\beta } e^{{i\phi_{k,\beta } }} + \ldots } \right] $$

where *D*_*k*,*α*_ is the number of connections between node *k* and module *α*, *R*_*k,α*_ stands for the influence module *α* has on the frequency of node *k*, and *ϕ*_*k*,*α*_ is the mean phase of module *α* nodes that are connected to node *k*. A similar expression can be used to describe the influence of hub nodes’ on the frequency of node *k*:

12 $$ R_{k,hubs} e^{{i\phi_{k,\,hubs} }} = \frac{1}{{D_{k,\,hubs} }}\sum\nolimits_{j \in hubs} {W_{kj} e^{{i\theta_{j} }} } $$

If node *k* is connected to only one node of module *α*, *D*_*k,α*_ = 1, it follows that *R*_*k,α*_ = 1 which cannot be interpreted as a measure of phase coherence. Therefore analyses were based on instances of *D*_*k,α*_ > 1.

As a measure of the impact of module nodes or hub nodes on nodes in another module, values of *R*_*k,α*_ were averaged over all nodes *k* belonging to the collection of nodes of interest, e.g.:

13 $$ R_{\beta ,\alpha } = \frac{1}{{N_{\beta } }}\mathop \sum \nolimits_{k \in \beta } R_{k,\alpha } $$

similarly, for hubs:

14 $$ R_{\beta ,hubs} = \frac{1}{{N_{\beta } }}\sum\nolimits_{k \in \beta } {R_{k,hubs} } $$

where *R*_*β,α*_ (or *R*_*β,hubs*_) denotes the average influence of module *α* nodes (or hub nodes) on the frequencies of module *β* nodes.

### Connectivity suppression

To assess the effects of suppressed connectivity on synchrony, selected edges were computationally removed from the adjacency matrix [42]. Hub connectivity was suppressed by decoupling all hub nodes with respect to each other. Since this type of hub connectivity suppression alters the overall connectivity density (which may have general consequences for network synchronization), an equal number of random edges was removed for reference purposes in the test condition. For trials with suppressed connectivity, the structural adjacency network, *W*, was adjusted by,

15 $$ W_{m,n \in K} = 0 $$

where *K* is the subset of nodes selected to be modulated. It is important to note that in the modulation conditions all hub nodes remained connected to the rest of the network as normal.

### Nodal frequency perturbation

In the trials where oscillator frequency was tracked, the data was grouped by module and a Fourier transform was applied in order to find the most powerful frequency within the modules. In the model trials, in which a set of nodes (i.e. hubs, random nodes or all nodes belonging to one of the modules) had their internal frequencies perturbed, the nodes were first assigned a random initial phase and internal frequency (as described above). Then, nodes within the selected set had their angular frequency increased by one.

To test the variation among dominant frequencies of the functional modules, a Bartlett’s test was performed on the unperturbed modules at the cortical coupling factor immediately prior to when the perturbed set of nodes became synchronized with the rest of the modules. For this purpose, whole brain synchronization was defined as the point at which lowest modular frequency was within 5 % of the highest modular frequency.

## Results

### Global synchronization

Assessing global synchronization in the anatomical brain network with the Kuramoto model revealed a modular state for low cortical coupling strengths, *λ*, that quickly transitioned into whole brain synchrony at increased cortical coupling. The level of global synchronization was measured by the Kuramoto model’s order parameters *r* and *r*_link_, denoting the extent to which nodes are in phase and the share of in-phase nodes respectively, where *r* (or *r*_link_) close to zero indicates global incoherence and *r* (or *r*_link_) near one reflects a state of global synchrony.

**Fig. 2:**
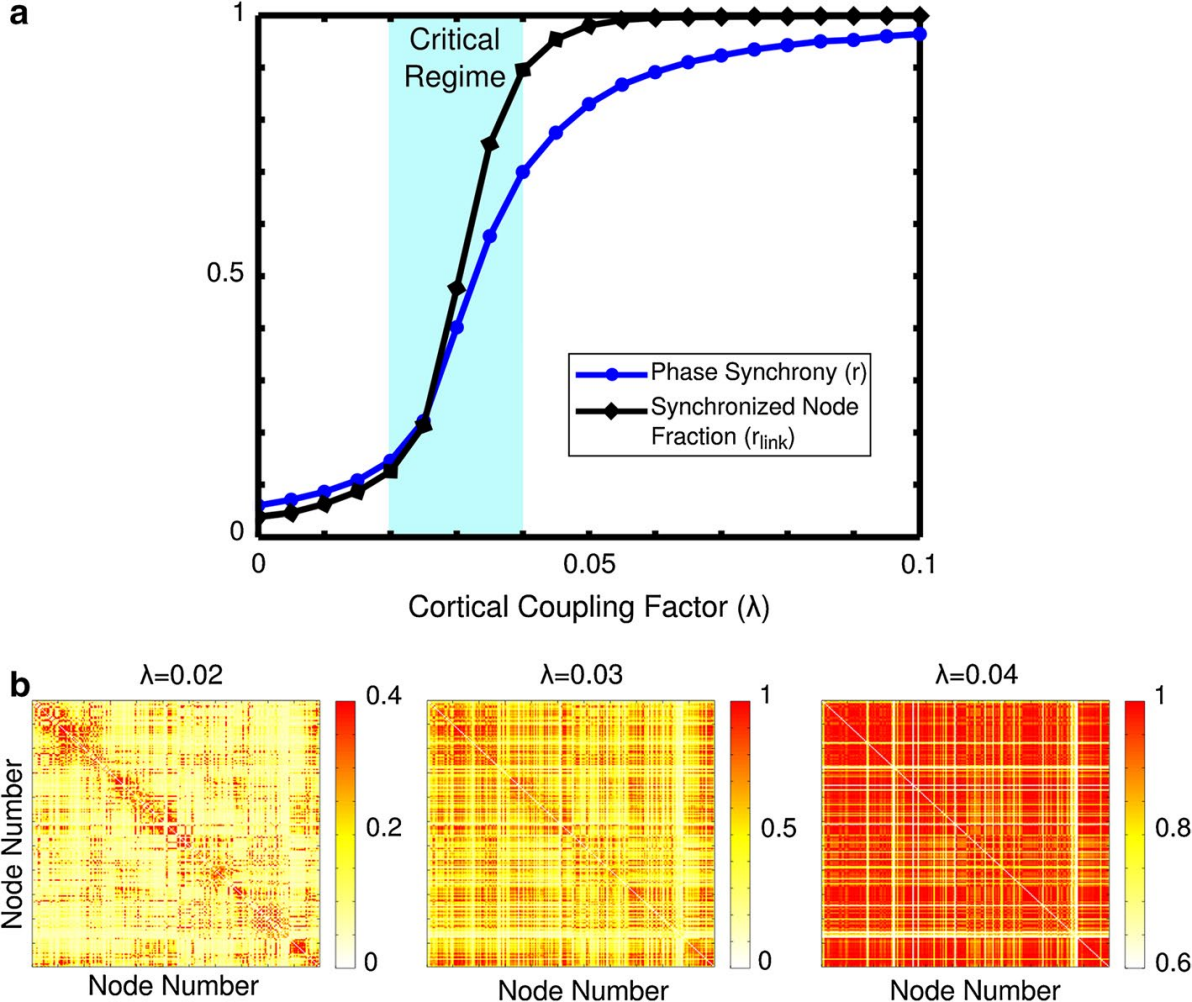
Global synchrony progression. The order parameters, *r,* reflecting global phase synchronization, and *r*_link_, reflecting the synchronized node fraction, increase with the coupling factor, *λ* (**a**). The critical regime, highlighted in *blue*, is the domain during which the system quickly transitions from the modular state to whole brain synchrony. *Panel* **b** shows the simulated functional synchrony output of the model at several different cortical coupling factors. A notable change from synchrony within the modules (*along the diagonal*) to whole brain synchrony occurs as the coupling factor is increased. Note that for illustrative purposes color scales vary among the three realizations of simulated functional synchrony

Figure 2a shows how levels of global synchronization progress with respect to cortical coupling strength. The order parameter *r* has been shown to follow a square-root function of the coupling strength *λ* [21], therefore the shape of the curve results from a square-root curve smoothed out by the heterogeneity in the intrinsic frequencies and by the network topology of the oscillators. A critical regime was observed between *λ* = 0.02 and *λ* = 0.04, indicated in blue in Fig. 2a, during which the network abruptly switches from brain regions being synchronized locally to a state of global synchrony, consistent with previous work on the cat cerebral cortex [3]. Figure 2b shows three panels of synchronization levels, or simulated functional connectivity, illustrating the modular state (small clusters of synchronized nodes), transition state (widespread synchrony arising) and global synchrony state (nearly all nodes in synchrony), evaluated by the Kuramoto model at cortical coupling factors *λ* = 0.02, *λ* = 0.03 and *λ* = 0.04, respectively.

### Network connectivity

Overlaying the reconstructed structural brain network, described by a binary 219 × 219 matrix derived from diffusion weighted imaging (DWI) data of 40 healthy subjects, with 11 commonly reported functional modules (see “Methods”), revealed that functional modules were structurally more densely connected as compared to the overall density of the SC matrix (Fig. 1, *p* < 10^−3^, permutation test, 10^3^ random selections of nodes). As a result, the highly internally connected functional modules produced higher levels of synchrony in our Kuramoto simulations, peaking at *λ* = 0.02. Synchrony was found to be significantly higher among node pairs that were directly connected by a structural connection compared with structurally unconnected node pairs (factor 3.9, *p* < 10^−4^, permutation test, 10^4^ random selections of unconnected node pairs), reflecting the apparent overlap between the lower triangular part of the matrix of Fig. 1, the SC matrix, and the upper triangular part containing the simulated synchronization levels. The high structural density of the functionally defined modules and their high levels of synchronization are thus indicative of a positive structure–function relationship, in accordance with recent literature [43–45].

### Hub selection and participation

The anatomical brain network showed a clear rich club organization [2, 20, 46], with nodes of high degree, *k*, or hub nodes, being more densely interconnected than expected by chance (11 < *k* < 44). Next, using *k* = 26, we selected the 39 nodes (18 % of all nodes) having the largest number of connections (degree), as the set of anatomical hubs [2, 3, 36]. These hub nodes were found to be distributed throughout the brain network and to participate in all 11 functional modules (Fig. 1), in accordance with the central embedding of hubs in the anatomical network and their suggested ability to facilitate communication and integration between anatomically segregated brain regions [3, 16].

### Modular synchronization

**Fig. 3:**
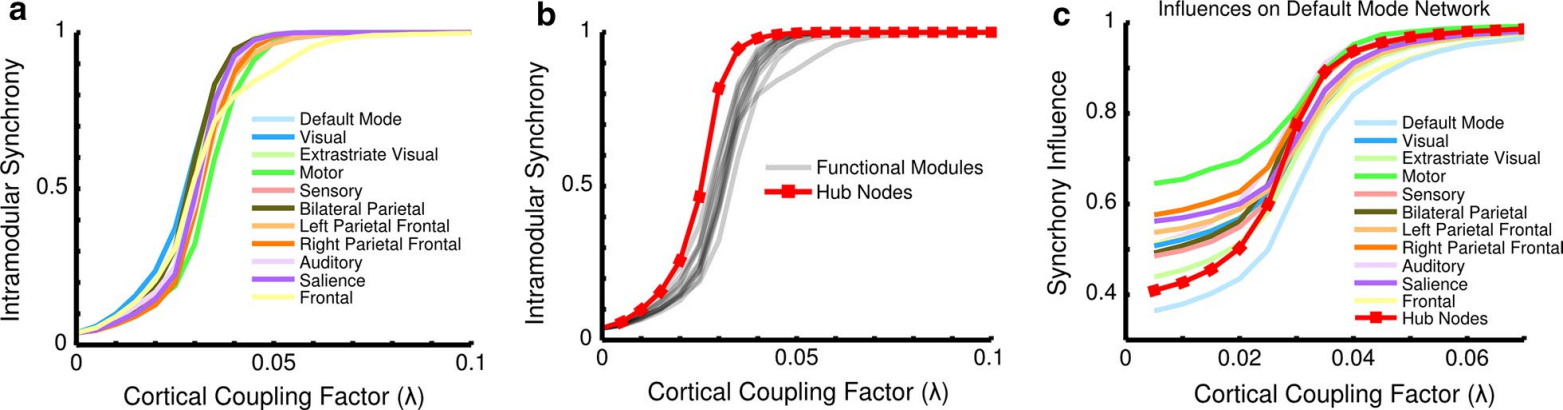
Intramodular synchrony progression. *Panel* **a** shows the evolution of intramodular synchrony within each of the 11 functional modules. The aberration of the Frontal module near whole brain synchrony is due to a single low-degree node. *Panel* **b** compares the intramodular synchrony of the modules to that of the hub nodes. Notably, the hub nodes led all of the functional modules in intramodular synchrony even though they were spatially distributed across the functional modules, and possessed a structural density on par with the average of the functional modules. *Panel* **c** shows the influence of each of the 11 modules and the hub nodes on the frequency of Default Mode Network nodes. The hub nodes become dominant in the process of global synchronization during the critical regime

Intramodular synchrony, reflecting the share of in-phase nodes within a functional module, was observed to evolve as an s-curve with respect to coupling strength in all 11 functional modules (Fig. 3a) again with a critical regime between *λ* = 0.02 and *λ* = 0.04 showing a steep increase in intramodular synchrony similar to global synchrony (Fig. 2). In Fig. 3b the synchronization among the hub nodes (intra-hub synchrony) is contrasted with the intramodular synchrony levels observed in each of the 11 functional modules. While similar in shape, the curve corresponding to the hub nodes is observed to be shifted towards lower cortical coupling strengths with respect to the graphs of the functional modules. In other words, intra-hub synchrony is higher than any module’s intramodular synchrony (*p* < 10^−4^, 10^4^ random permutations of connection labels ‘intra-hub’ and ‘intramodular’, 0.02 < *λ* < 0.05), implying that the rich club structure, spread out across the brain network and involved in all functional modules, requires less cortical coupling in order to reach synchrony than the similarly dense or even denser functional modules.

Furthermore, nodes participating in the same functional module were found to be more closely synchronized to each other than nodes from different modules, reflected by a 1.5 times higher synchrony between nodes within a module than between nodes in different modules (*p* < 10^−3^, 10^3^ random permutations of connection labels ‘intramodular’ and ‘intermodular’, evaluated at the onset of the critical regime, *λ* = 0.02). Examining hubs showed this class of nodes to have a significantly higher synchronization among themselves than the nodes belonging to any functional module or than an equally sized random set of nodes (*p* < 10^−4^, 10^4^ random permutations of node labels ‘hub’ and ‘modular’, 0.005 < *λ* < 0.075), indicating that global hub nodes are leading the functional modules in establishing local modular synchrony. In addition, synchrony between a functional module and the set of hub nodes was found to be significantly higher than intermodular synchrony (*p* < l0^−4^, 10^4^ random permutations of connection labels ‘hub-module’ and ‘intermodular’) for 53 out of all 55 combinations of functional modules (11 × 10/2) (Additional file 2: Figure S2), suggesting the structure of the network supports intermodular synchronization through the strongly tied hub nodes.

The mean field representation of the Kuramoto model and the break down of synchrony contributions across the functional modules and the hub nodes Eqs. 13 and 14, allowed for the comparison between the contributions of modules and hub nodes towards global synchrony. Figure 3c shows the synchrony contributions received by the Default Mode Network from each of the functional modules and the hub nodes (for the other modules see Additional file 3: Figure S3). In the critical regime between λ = 0.02 and λ = 0.04, where the hub nodes become more synchronized among each other, the hubs exert stronger influence on the modules and take on a dominant role in exerting influence on the rest of the network, evidenced by the steepest increase in impact on functional modules compared to the change in influences exerted by the modules (Additional file 3: Figure S3, paired *t*-test between a module’s and the hub nodes’ impact on the set of 11 modules, p < 0.02). Moreover, at the end of the critical regime, λ = 0.04, the hub nodes’ influence on each of the modules is greater than that of 9 out of 11 modules (paired *t*-test, p < 0.04) and not significantly different from the two remaining modules (Visual and Bilateral Parietal, paired *t*-test, p > 0.85).

### Hub connectivity suppression

**Fig. 4:**
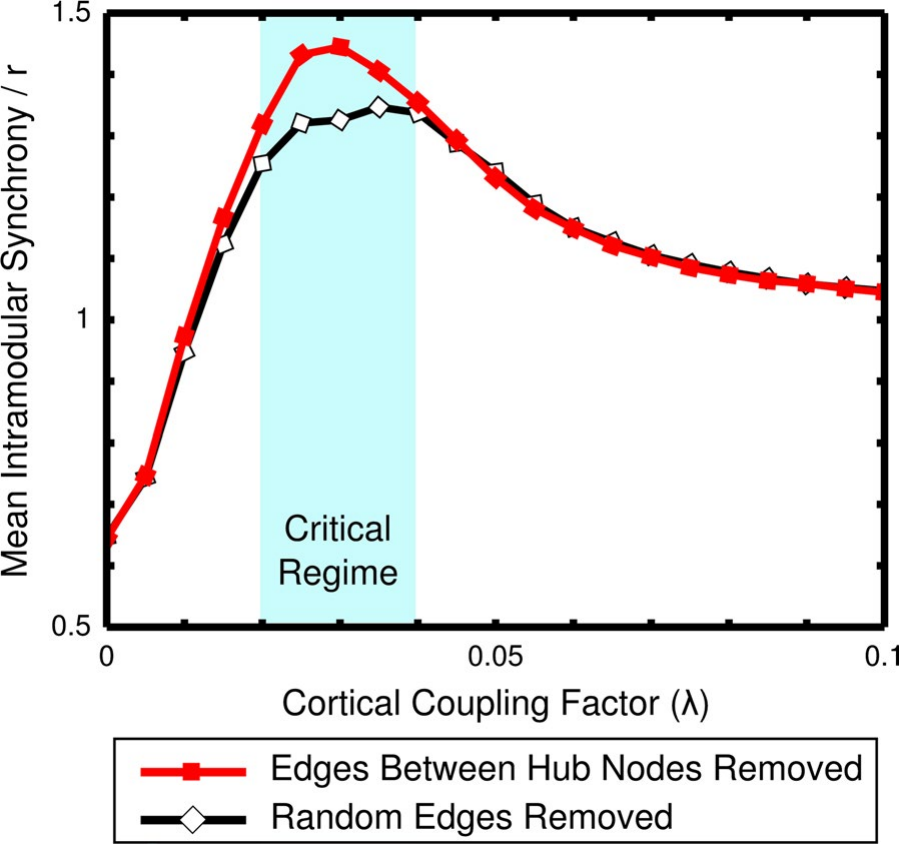
Modularity increased with hub connectivity suppressed. The ratio between the average intramodular synchrony of the functional modules and the whole brain synchrony is displayed for the cases in which either edges between hub nodes or random edges have been removed. Interestingly, when hub connectivity was suppressed, intramodular synchrony relative to global synchrony increased during the critical regime. Conversely, this increase in modularity was not observed when only random edges were removed

Next, we examined the role of hub nodes in establishing global synchrony. Targeted suppression of structural connectivity was inflicted by computationally removing edges between hub nodes. For comparison, in a separate simulation, an equal number of random edges were removed. In simulations with suppressed connectivity (either between hubs or between random nodes), stronger cortical coupling was required for synchronization to compensate for the loss of connectivity, while the general shape of global synchronization progression towards a state of global synchrony remained the same (Additional file 4: Figure S4). However, simulations in which hub-to-hub connectivity was suppressed showed a significantly higher intramodular to global synchrony ratio than randomly suppressed connectivity simulations (Fig. 4) (*p* < 4.1 × l0^−3^, 10^4^ random permutations of hub-to-hub and random connectivity suppressions, 0.015 < *λ* < 0.04). This increased intramodular to global synchrony ratio as seen in the hub suppressed network indicates that connections spanning between hub nodes (so-called ‘rich club connections’ [29]) play an important role in the integration of modules needed to reach global synchrony. Also in comparison with the unsuppressed, normal condition, suppression of hubs resulted in increased modularity (Additional file 5: Figure S5), again illustrating that without interconnected hub nodes, functional modules become more separated and the emergence of global synchrony is hampered.

### Hub perturbation

**Fig. 5:**
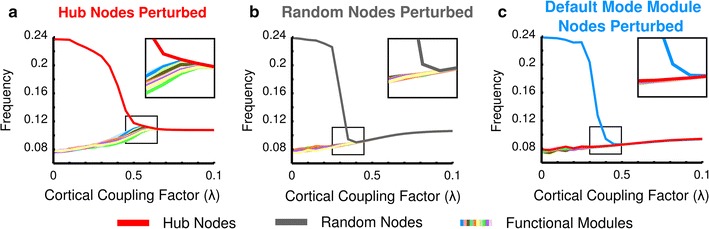
Modular frequency tracking during perturbation. When the internal frequencies of a particular module were perturbed, the evolution of the frequencies towards whole brain synchrony was tracked for each module. *Panel* **a** shows the case in which the hub nodes had their internal frequencies altered, and the rest of the modules were unable synchronize until the hub nodes’ frequency came down to the range of frequencies of the functional modules. *Panel* **b** shows the case in which a random set of nodes equal in number to the rich club has been perturbed. Here, synchronization occurred faster than when the hub nodes were perturbed, and the functional modules were able to synchronize before the random nodes join at a whole brain shared frequency. *Panel* **c** shows the case in which the largest functional module (Default Mode) was altered. Note that, in this case too, the rest of the modules are able to synchronize before the Default Mode module joins. The *insets* in each graph focus on the cortical coupling factors just before the perturbed set of nodes became synchronized with the unperturbed functional modules. From these insets, it is clear that perturbing the hub nodes causes less synchrony among the non-perturbed functional modules

Apart from manipulating connectivity through edges as presented in the previous analysis, synchronization dynamics in the Kuramoto model can also be perturbed by offsetting the initial internal frequencies of a set of nodes. As lower levels of synchronization are a trivial consequence of these frequency perturbations, we tracked nodal frequencies along the path towards global synchrony to examine how different sets of nodes affected the synchronization of the entire system. Figure 5a shows that with the frequency of the hub nodes perturbed, the functional modules remained out of synchrony with respect to each other until the hub nodes stabilized at a frequency near the whole brain frequency corresponding to global synchrony. In contrast, in all other simulations where a different set of nodes was perturbed, the frequencies of the unperturbed modules converged to a common global frequency, reflecting global synchronization, even though the perturbed set of nodes were still operating at a markedly higher frequency. For example, when a random set of nodes (equal in number to the hub nodes) had their internal frequencies perturbed, the remaining unperturbed portion of the system was found to synchronize without the perturbed set of nodes (Fig. 5b). Similarly, perturbing the nodes of any of the 11 functional modules did not keep the other modules from synchronizing among one another (see Fig. 5c for an example in which the Default Mode Network module was perturbed). Nearly identical findings for the remaining 10 functional modules are provided in Additional file 6: Figure S6.

Testing the variation of frequencies among modules in the case of hub node perturbation, compared to if either a functional module or a random set of nodes was perturbed, showed, as depicted in Fig. 5, that hub node perturbation caused the highest frequency variation among the functional modules (*p* < 6× 10^−9^, Bartlett’s test). This effect of relative global asynchrony with perturbed hub nodes remained present when a smaller rich club comprising 22 hub nodes, closer to the average functional module size of 17. 9 ± 5.1 nodes, was examined (Additional file 7: Figure S7). Taken together, these frequency perturbation findings suggest that the hub nodes are essential components that cannot operate at frequencies isolated from the rest of the network in order for global synchronization to occur.

### Macaque verification

A reconstruction of the macaque connectome, based on an extraction from the CoCoMac database [37] containing anatomical tract-tracing data (see Methods), was used to verify synchronization effects observed in the human cortical network. Analogous to the main analysis of the human cortical network, identical Kuramoto simulations were run on the SC data describing the macaque connectome, of which the results are summarized below. Findings in the macaque were in close agreement with those in the human:

*Global synchrony* Global synchronization in the macaque brain was observed to increase with cortical coupling strength and revealed a modular state and a state of global synchrony separated by a critical regime (Additional file 8: Figure S8).

*Network connectivity* Synchrony between structurally connected nodes was higher than synchrony between nodes not directly connected; the ratio between these two values peaked early in the critical regime at λ = 0.015, where synchrony in directly structurally connected nodes was 3.07 times higher (p < 10^−5^).

*Hub selection and participation* Rich club organization was found for *k* > 14 to *k* = 56. The 15 nodes (19 %) having degree *k* > 38 were selected as the set of hub nodes. These hubs were again found to be distributed across the cortical network, participating in all functional modules.

*Modular synchronization* The synchronization among hub nodes and the intramodular synchronization showed the characteristic s-curve progression for increasing cortical coupling strength, with the hub nodes synchronizing at lower cortical coupling than the modules. Furthermore, synchrony between a module and the hub nodes during the modular state (*λ* = 0.01) was found to be significantly higher than intramodular synchrony for all 6 out of 6 modules and higher than intermodular synchrony (*p* < 7.2 × 10^−4^) for all 15 combinations of modules (6 × 5/2).

Hubs showed a steeper increase in their influence on the frequencies of modules during the critical regime than 4 out of 6 modules (paired *t*-test, p < 0.03) and at the end of the critical regime, the hubs’ impact on each of the modules is greater than that of all 6 modules (paired *t*-test, p < 0.04).

**Fig. 6:**
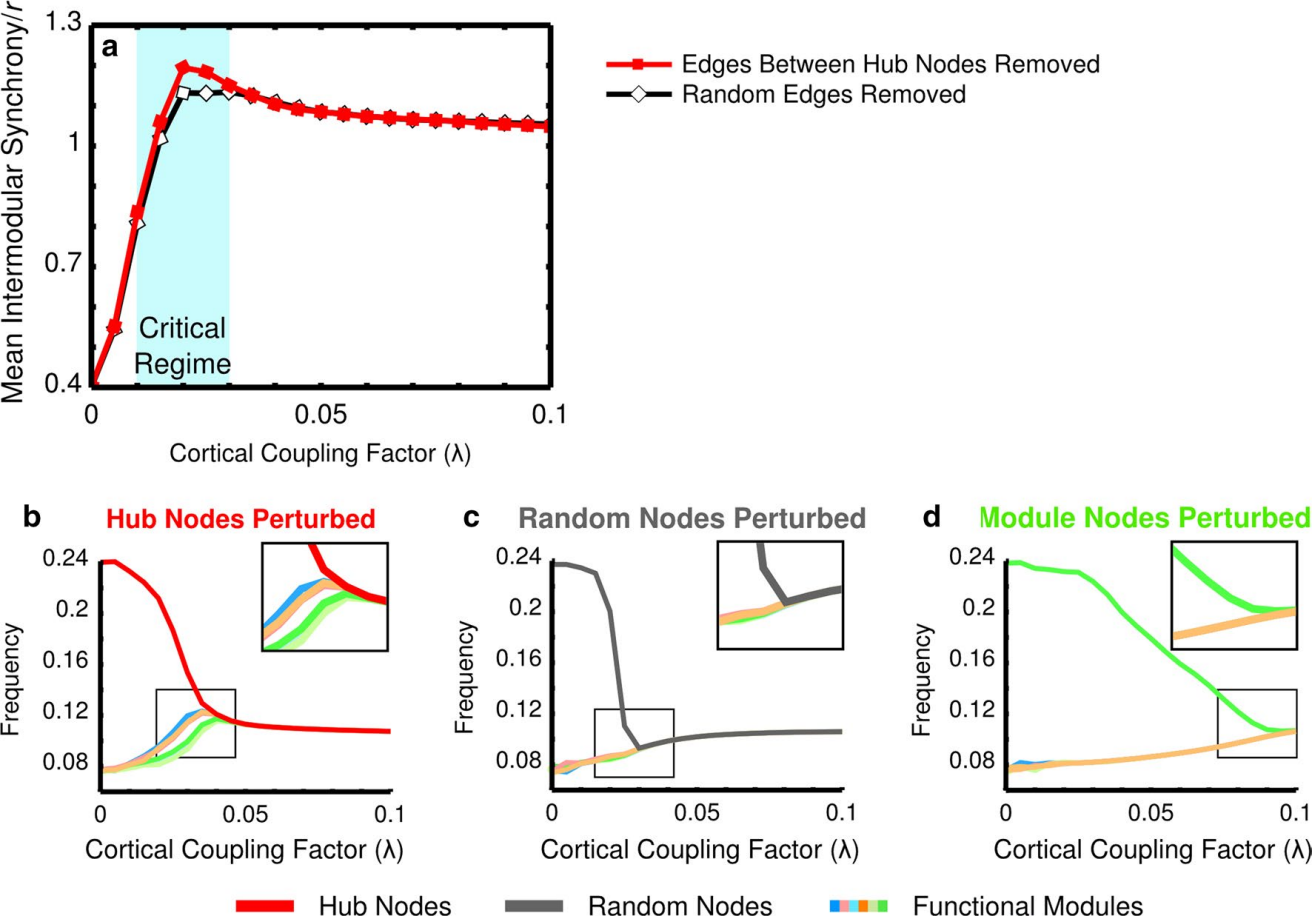
Macaque verification of hub connectivity suppression and perturbation effects. Suppressing hub connectivity in the macaque cortical network verified the result of increased modularity found in the human cerebral cortex network (Fig. 4). Throughout the critical regime modules were found to have increased intramodular synchrony when hub connectivity was suppressed pointing at the important role of hub nodes in establishing intermodular and global synchrony (**a**). Similar to the effects observed in the human network (Fig. 5), perturbing the internal frequencies of the hub nodes in the macaque cortical network prevented the modules from synchronizing (**b**). Perturbation of an equally large random set of nodes (**c**) or a module (**d**) did not keep the unperturbed part of the network from synchronizing, underlining the importance of the hub nodes in intermodular communication and integration

*Hub connectivity suppression* Simulations in which hub connectivity was suppressed showed increased modularity (higher intramodular to global synchrony ratio, *p* < 10^−4^, 0.01 < *λ* < 0.03) compared with the removal of an equal sized random set of connections (Fig. 6a).

*Hub perturbation* Perturbation of the hub nodes revealed that the remaining unperturbed nodes of the network were not able to synchronize before the hub nodes had converged to the frequency of global synchrony (Fig. 6b). In contrast, for a perturbed random set of nodes or a perturbed module, the unperturbed part of the network was still able to synchronize (Fig. 6c, d). Significantly higher variation was observed for the hub nodes perturbed simulations than if a random set of nodes or a module was perturbed (*p* < 7 × 10^−5^).

## Discussion

Our simulations of synchrony patterns based on a structural reconstruction of the human cortical network produce converging evidence that hub nodes act as spatially distributed but functionally central integrators of neural information between otherwise segregated functional domains. First, hub nodes showed high levels of synchronization among themselves and were found to be distributed across all functional modules. Second, suppression of connectivity among hub nodes caused the network to show stronger modularity in synchronization patterns. Third, perturbation of hub nodes’ intrinsic oscillatory behavior prevented the functional modules from synchronizing.

We verified these findings in the macaque, using a connectome reconstruction based on collated tract-tracing data.

Functional modules based on resting-state fMRI data displayed high structural density as well as strong intramodular synchrony, providing simulation based indications of a positive structure–function relationship in empirical data [29, 44, 47].

Previous modeling work revealed that the wiring of cortical networks gives rise to the formation of functional modules [14, 48–50]. Our simulations over a range of coupling strengths showed a critical regime [3, 25] separating a modular state from the state of global synchrony. Although caution is needed to directly interpret the simulated cortical coupling strengths of cortico-cortical connections as a mechanism to allow a cortical network to switch between a modular state and global synchrony, our results do support the notion of topological organization of the cortical network to enable theoretical synchrony both at the modular and at the global level [51]. The state of global synchrony itself is not to be directly interpreted as biologically meaningful, being merely the attractor state of the Kuramoto model. It is the progression towards global synchrony that reveals how synchronization—and therewith hypothesized binding of information [10, 12]—among segregated brain regions may dynamically occur.

Hub nodes were found to be distributed across the cortical network and to participate in all functional modules, consistent with previous observations [16, 36, 44–46], which are characteristics well-suited to a facilitating role in intermodular communication and integration contributing to global synchrony [52]. In further support of the importance of hub nodes in global synchrony, our simulation results show synchronization among hub nodes to be stronger than the intramodular synchronization of the functional modules, suggesting that the network topology is such that hub nodes form a closely tied and functionally linked ensemble leading the functional modules in establishing synchrony. Anatomically densely intra-connected functional modules showed the highest levels of intramodular synchrony. Hub nodes stood out due to their strong mutual synchronization while having a structural density near the average density of the functional modules, indicating strong synchrony among hub nodes was not purely a consequence of structural density.

Our observation of strong synchrony between hub nodes aligns well with the results of two other Kuramoto simulation studies, one on the macaque cortex, reporting hubs to synchronize at shorter timescales compared to other nodes [22] and another on the cat cortex reporting anatomical hubs to be leading the transition to whole brain synchrony [3]. Moreover, a recent study of our group in which we used an alternative model for the simulation of brain dynamics (an Ising spin glass model [53]) showed the tendency of hub nodes to collectively be in an ‘activated state’ and to enrich the overall functional dynamics of the system [49].

Suppressing connectivity, irrespective of the targeted edges in the network, resulted in a reduction of global and intramodular synchrony, directly related to the sparser structural input. The ratio between intramodular and global synchrony provided more insight into the relative effect of connectivity suppression, with targeting the connections linking hub nodes leading to an increased intramodular to global synchrony ratio. Such an increase in modularity was not observed when a random set of connections was removed (Additional file 5: Figure S5), indicating connections between hub nodes to be particularly important for intermodular communication and integration.

Further evidence of the importance of hub nodes in shaping intermodular functional dynamics was demonstrated by perturbing the intrinsic oscillatory behavior of the hub nodes. While offsetting the internal frequencies of the hub nodes did not have a permanent effect on the network dynamics other than a trivial change in whole brain synchrony state frequency, the functional modules remained desynchronized until the hub nodes approached a matching synchronization frequency (Fig. 5a). Perturbing a functional module or an equal number of random nodes, by contrast, did not prevent the unperturbed modules from synchronizing with each other, leaving the perturbed set of nodes isolated (Fig. 5b, c).

An important consideration resulting from the interpretation of *r*_link_ as a fraction of synchronized nodes is that the cut-off value of *C*_*ij*_, above which node pairs are considered to be in synchrony, may vary. As a consequence, node pairs with a relatively high level of synchronization, falling just below the cut-off value, can be classified as completely incoherent using this approach. Interpreting *r*_link_ as the fraction of synchronized nodes is most accurate where there is a clear divide between node pairs in their levels of synchronization. Furthermore, it should be noted that a binary, undirected adjacency matrix was used as structural input to our simulations, resulting in each edge to contribute equally in the model drawing all nodes to a global frequency converging to the average of all internal node frequencies. Future studies could focus on modified Kuramoto model implementations, e.g. including connection weights, for enhanced biological plausibility [41] to further elucidate the observed patterns of modular and global synchronization. Another limitation arises from the crudeness of the suppression applied to the structural input. Reduced white matter integrity is linked to various psychiatric and neurological disorders, but at the scale of this cortical network, consisting of 219 nodes, disease effects including the loss of entire anatomical connections are improbable. It would therefore be of interest to explore the effects of connectivity suppression in a weighted approach, in which strengths of individual connections can be adjusted.

In the macaque verification analyses ‘functional’ modules were derived from anatomical connectivity. For consistency with our module definition in the human, macaque resting-state fMRI acquisitions would have been ideal. However, with structural and functional connectivity and modularity strongly overlapping both in the human [43, 54] and in the macaque [47, 55], the structurally defined modules are a valid approximation for macaque functional modules.

## Conclusion

The synchronization patterns examined in this study provide insight into how anatomical hub nodes may form an infrastructure facilitating communication between functional modules enabling global binding and integration of information.

The Kuramoto model’s ability to simulate structurally dependent functional synchrony could be further utilized to simulate neurological and neuropsychiatric diseases that affect the anatomical connectivity of the brain, and could potentially aid in understanding the relationships between disease structural abnormalities and changes in functional brain dynamics and connectivity.

## Additional files

Additional file 1:
**Figure S1.** Functional modules derived from resting-state fMRI data. This figure from Van den Heuvel and Sporns, 2013, shows the location of the functional modules based on independent component analysis (ICA) of resting-state fMRI data. Additional file 2:
**Figure S2.** Inter- and intramodular synchrony. Each bar plot corresponds to the inter- and intramodular synchrony of each of the 11 functional resting-state modules and the hub nodes. Shown synchronization levels were evaluated at the onset of the critical regime with a cortical coupling factor λ = 0.02. The hub nodes showed particularly high synchrony compared with the functional modules even though they were distributed across the modules. In some cases, synchrony between the hub nodes and a module was even higher than the module’s intramodular synchrony (see plot 1, 5, 6 and 8). This strong level of synchrony suggests the hub nodes’ importance for synchrony across the network. Additional file 3:
**Figure S3.** Influences on oscillation frequencies of the modules and hub nodes. For each of the 11 modules (1 plot per module), the influences on its frequencies of the modules and hub nodes (12 lines per plot corresponding to the modules and the hub nodes) are shown. The hub nodes become dominant in the process of global synchronization during the critical regime. Additional file 4:
**Figure S4.** Hub versus random connectivity suppression. When edges were removed from the adjacency matrix, attaining whole brain synchrony required a higher cortical coupling factor. Removing both random and edges between hub nodes produces this result. Additional file 5:
**Figure S5.** Modularity increased with hub connectivity suppressed. The ratio between intramodular synchrony and whole brain synchrony is shown for normal, hub connectivity suppressed, and random edge suppressed states. During the critical regime, the random edge suppressed state was observed to be almost identical to the normal state. In contrast, the hub connectivity suppressed state shows a significantly increased intramodular synchrony relative to whole brain synchrony, reflecting stronger modularity in the hub connectivity suppressed state. Additional file 6:
**Figure S6.** Modular frequency tracking during perturbation. Next to the Default Mode module (Figure 5), the effects of perturbation were examined for the remaining 10 functional modules as well. In each instance, the non-perturbed modules synchronized before the perturbed module joined in whole brain synchrony, consistent with the Default Mode perturbation and distinctly different from the hub node perturbation shown in figure 5. Additional file 7:
**Figure S7.** Small set of hub nodes perturbed. Perturbation of a smaller set of hub nodes also prevented the functional modules from synchronizing until the hub nodes’ frequency joined in whole brain synchrony. Additional file 8:
**Figure S8.** Global synchrony progression in the macaque. The order parameters *r* and *r*_link_ for the macaque model network progressed in a manner similar to the human network (Figure 2), displaying a modular and a whole brain synchrony state separated by a critical regime.

## Footnotes

1 *r*_link_ is equivalent to *r**_link_ in [3], in which the Kuramoto model was applied to the cat brain.
