# Supplementary figures and images for: Kuramoto model simulation of neural hubs and dynamic synchrony in the human cerebral connectome
Source: BMC Neurosci. 2015 Sep 2;16:54. doi: 10.1186/s12868-015-0193-z (PMC4556019; doi:10.1186/s12868-015-0193-z)

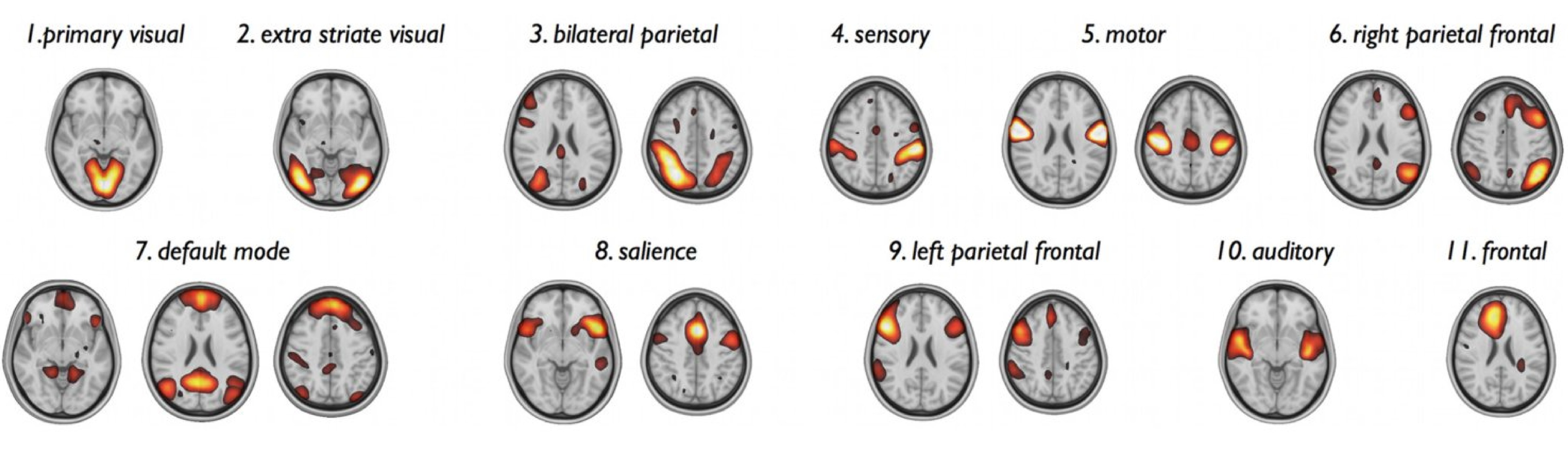

Supplement: Additional file 1: — Figure S1. Functional modules derived from resting-state fMRI data. This figure from Van den Heuvel and Sporns, 2013, shows the location of the functional modules based on independent component analysis (ICA) of resting-state fMRI data. [file 12868_2015_193_MOESM1_ESM.tiff]

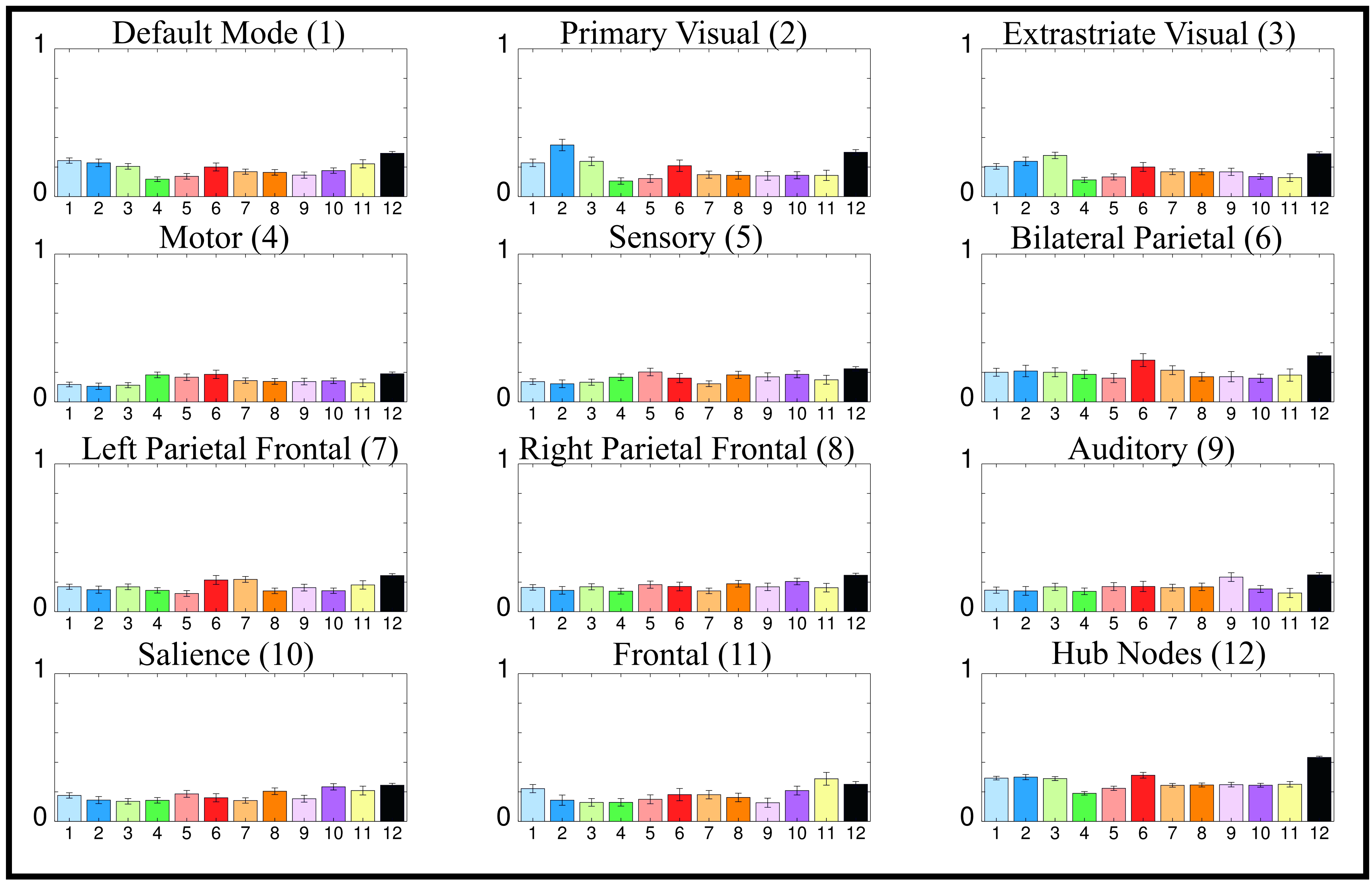

Supplement: Additional file 2: — Figure S2. Inter- and intramodular synchrony. Each bar plot corresponds to the inter- and intramodular synchrony of each of the 11 functional resting-state modules and the hub nodes. Shown synchronization levels were evaluated at the onset of the critical regime with a cortical coupling factor λ = 0.02. The hub nodes showed particularly high synchrony compared with the functional modules even though they were distributed across the modules. In some cases, synchrony between the hub nodes and a module was even higher than the module’s intramodular synchrony (see plot 1, 5, 6 and 8). This strong level of synchrony suggests the hub nodes’ importance for synchrony across the network. [file 12868_2015_193_MOESM2_ESM.tiff]

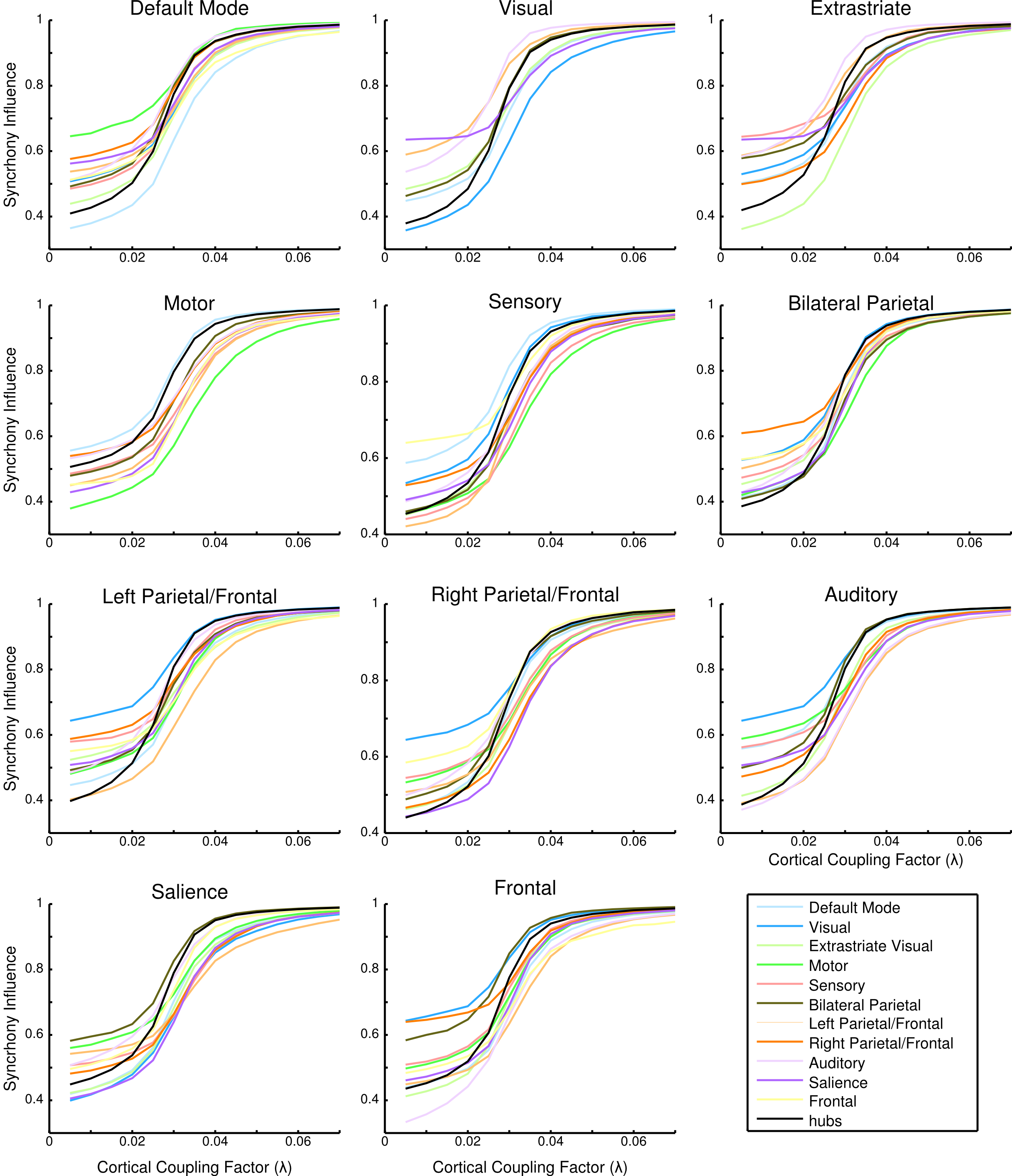

Supplement: Additional file 3: — Figure S3. Influences on oscillation frequencies of the modules and hub nodes. For each of the 11 modules (1 plot per module), the influences on its frequencies of the modules and hub nodes (12 lines per plot corresponding to the modules and the hub nodes) are shown. The hub nodes become dominant in the process of global synchronization during the critical regime. [file 12868_2015_193_MOESM3_ESM.tiff]

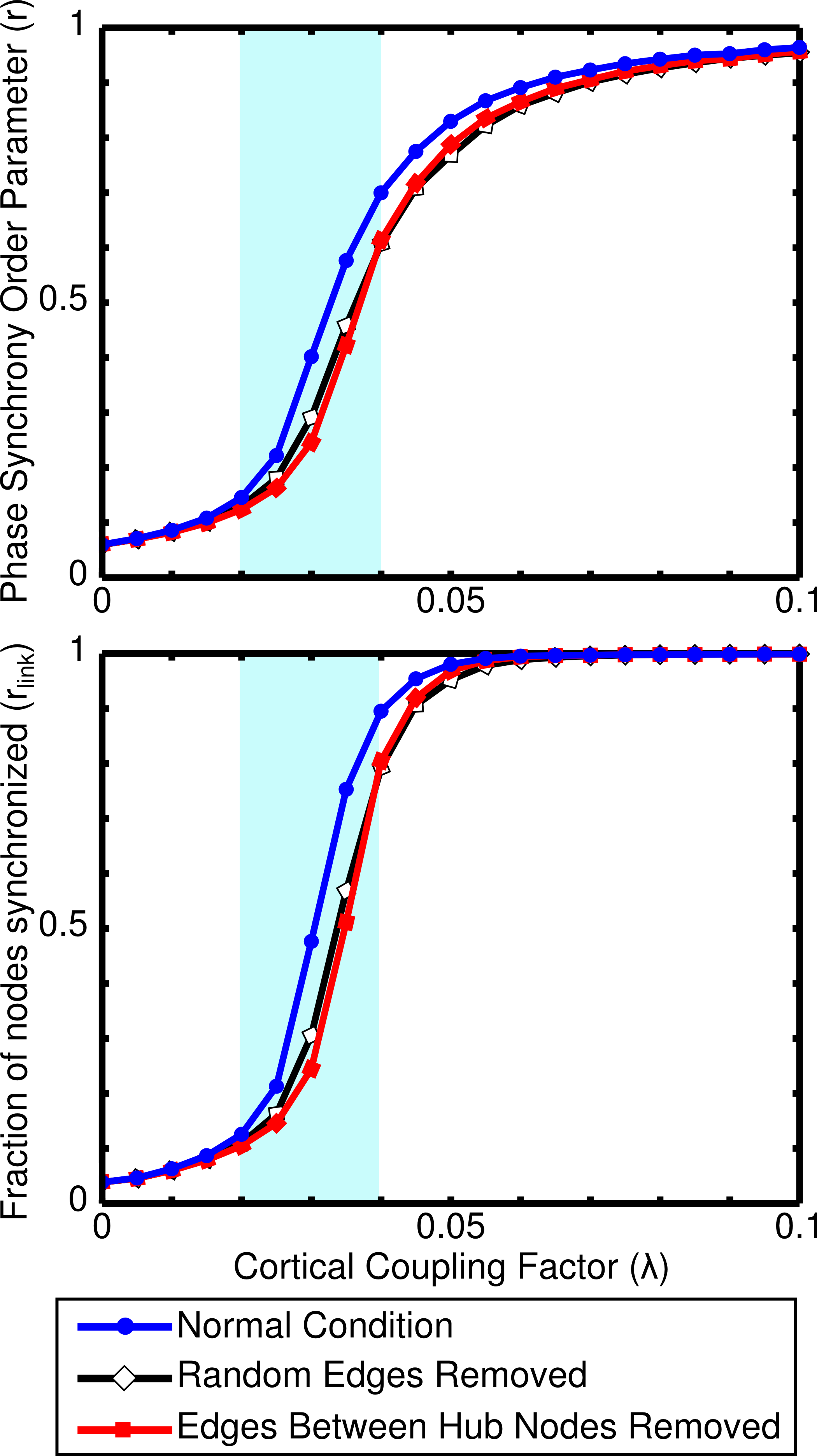

Supplement: Additional file 4: — Figure S4. Hub versus random connectivity suppression. When edges were removed from the adjacency matrix, attaining whole brain synchrony required a higher cortical coupling factor. Removing both random and edges between hub nodes produces this result. [file 12868_2015_193_MOESM4_ESM.tiff]

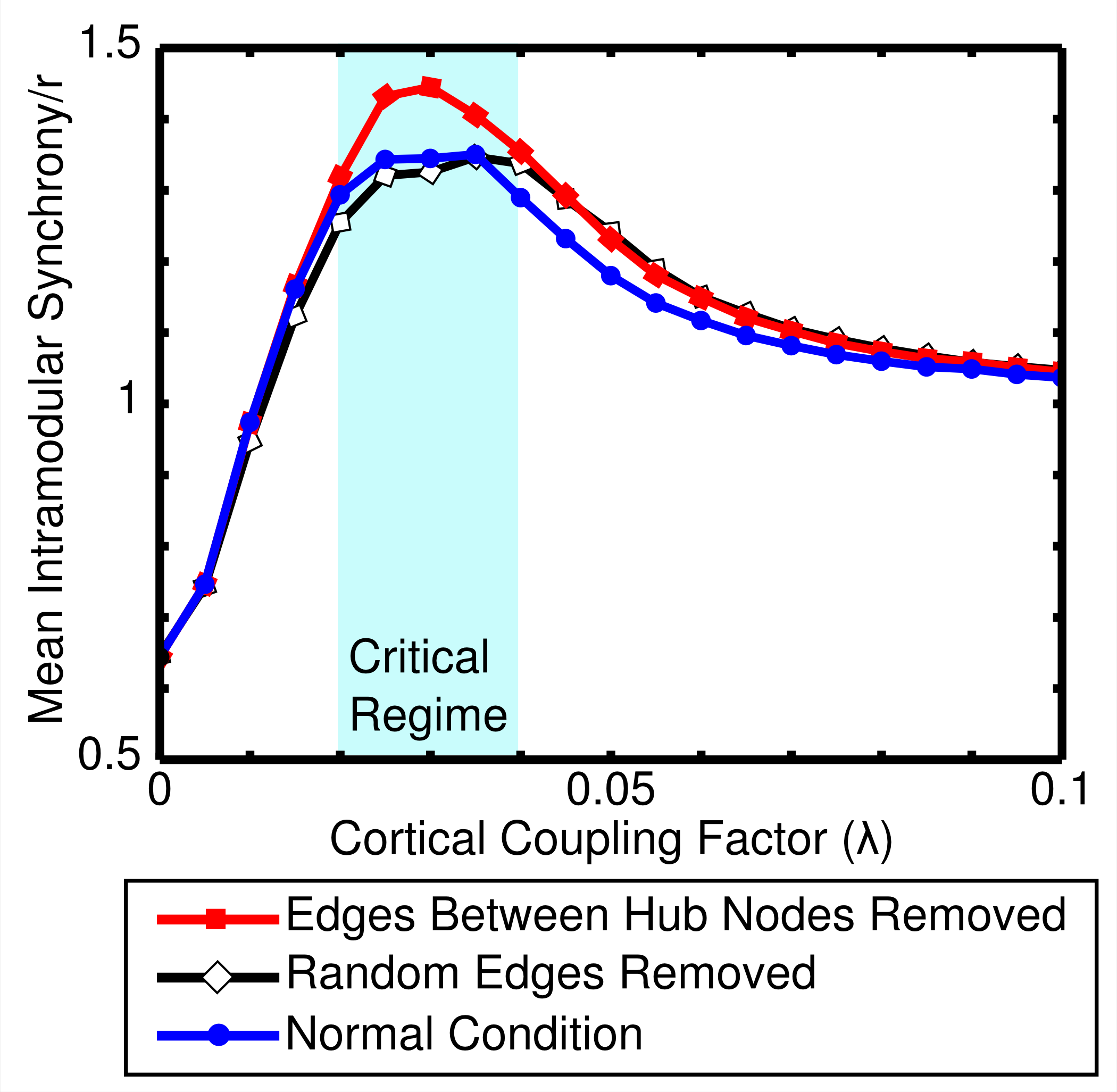

Supplement: Additional file 5: — Figure S5. Modularity increased with hub connectivity suppressed. The ratio between intramodular synchrony and whole brain synchrony is shown for normal, hub connectivity suppressed, and random edge suppressed states. During the critical regime, the random edge suppressed state was observed to be almost identical to the normal state. In contrast, the hub connectivity suppressed state shows a significantly increased intramodular synchrony relative to whole brain synchrony, reflecting stronger modularity in the hub connectivity suppressed state. [file 12868_2015_193_MOESM5_ESM.tiff]

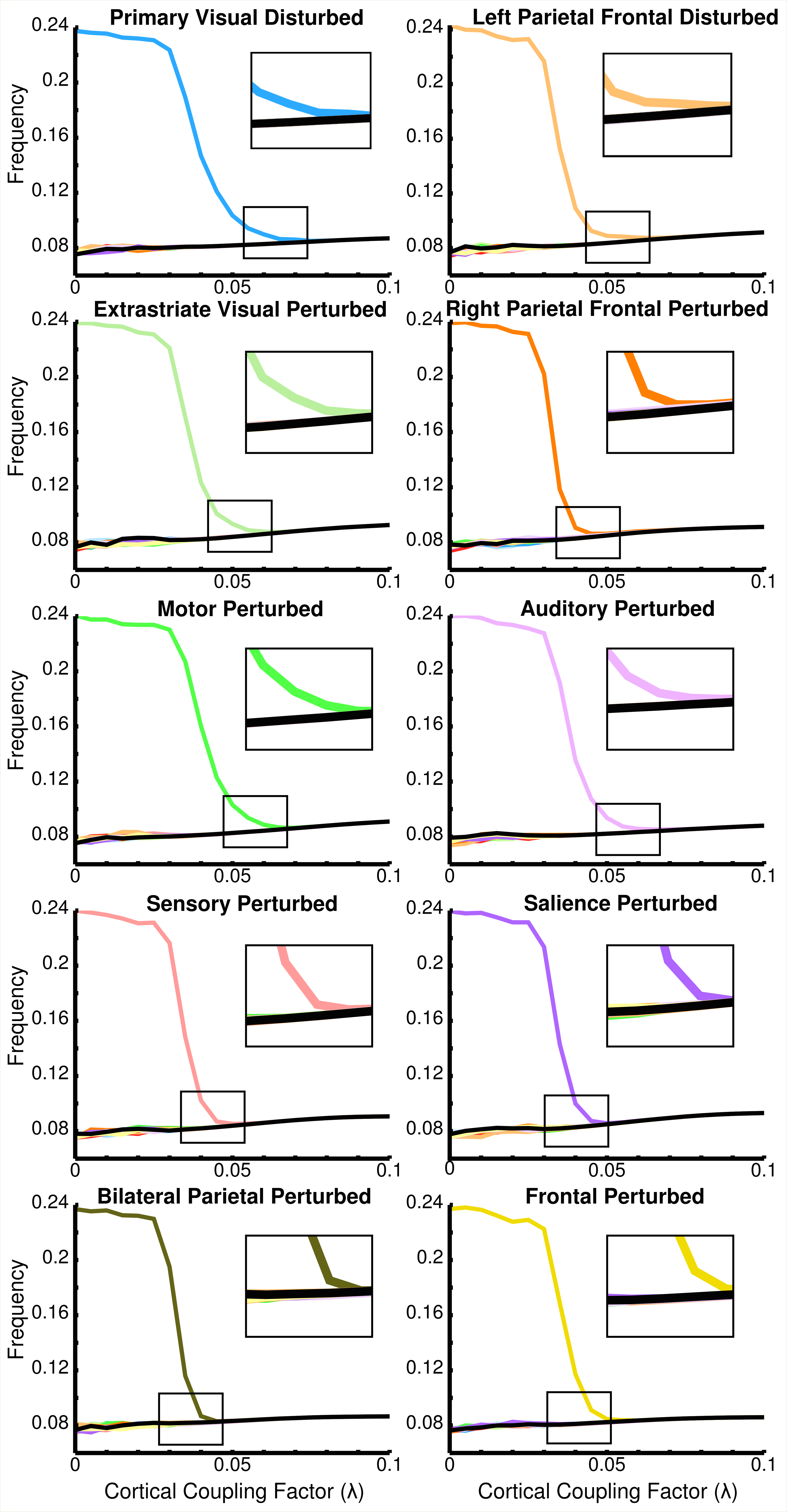

Supplement: Additional file 6: — Figure S6. Modular frequency tracking during perturbation. Next to the Default Mode module (Figure 5), the effects of perturbation were examined for the remaining 10 functional modules as well. In each instance, the non-perturbed modules synchronized before the perturbed module joined in whole brain synchrony, consistent with the Default Mode perturbation and distinctly different from the hub node perturbation shown in figure 5. [file 12868_2015_193_MOESM6_ESM.tiff]

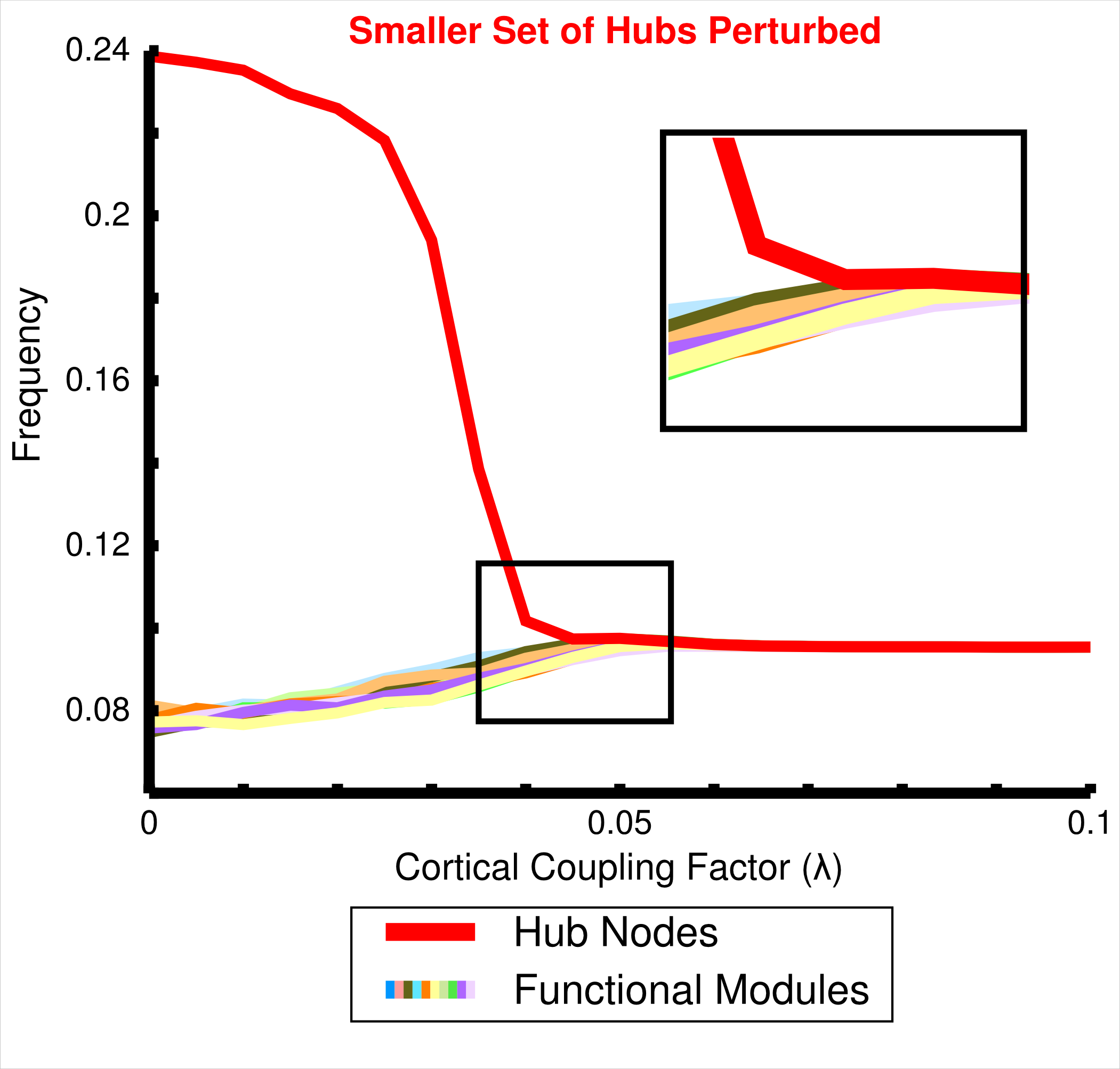

Supplement: Additional file 7: — Figure S7. Small set of hub nodes perturbed. Perturbation of a smaller set of hub nodes also prevented the functional modules from synchronizing until the hub nodes’ frequency joined in whole brain synchrony. [file 12868_2015_193_MOESM7_ESM.tiff]

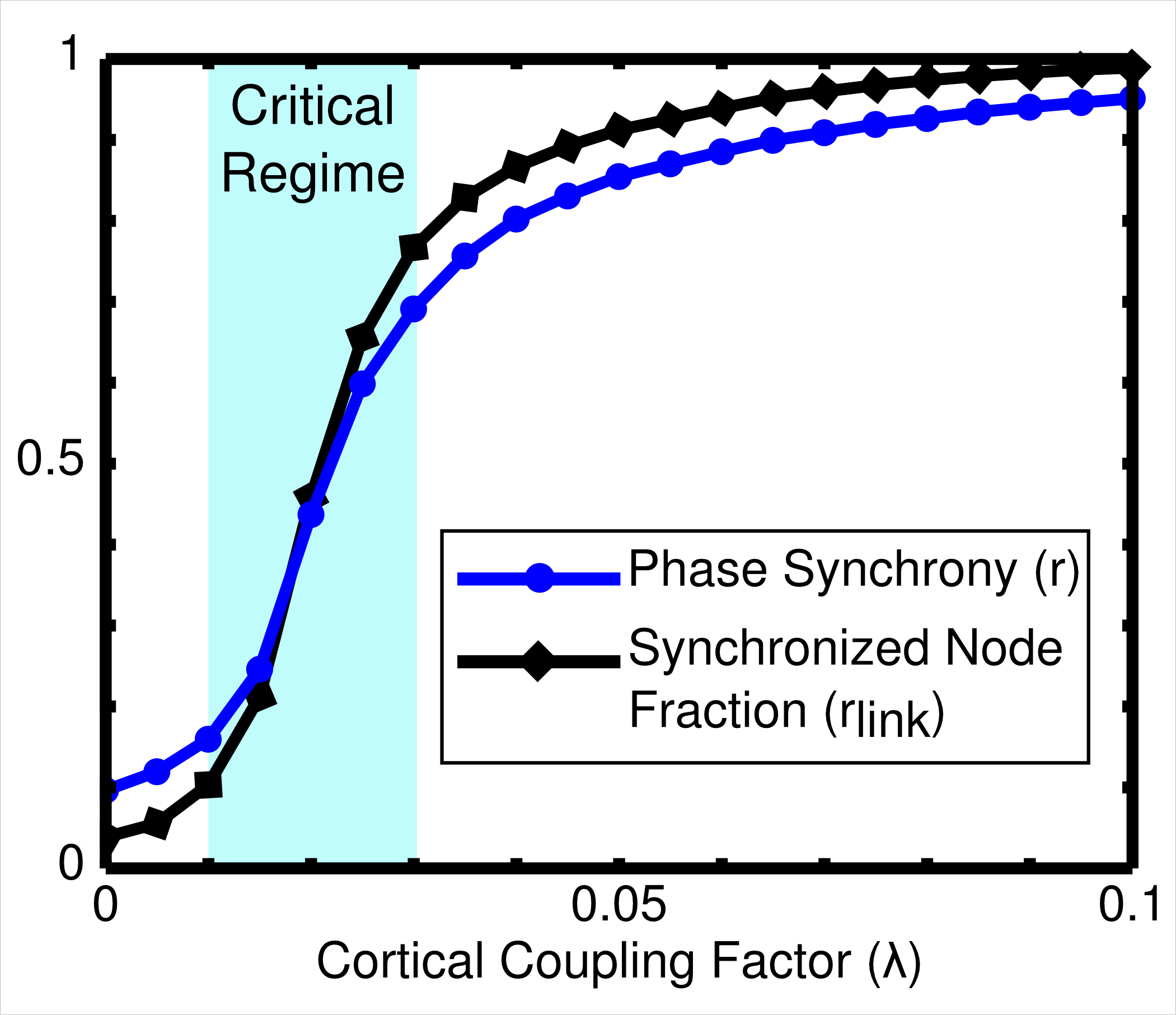

Supplement: Additional file 8: — Figure S8. Global synchrony progression in the macaque. The order parameters r and r link for the macaque model network progressed in a manner similar to the human network (Figure 2), displaying a modular and a whole brain synchrony state separated by a critical regime. [file 12868_2015_193_MOESM8_ESM.tiff]
